# Prostate Cancer in 2021: Novelties in Prognostic and Therapeutic Biomarker Evaluation

**DOI:** 10.3390/cancers13143471

**Published:** 2021-07-11

**Authors:** Alessia Cimadamore, Roberta Mazzucchelli, Antonio Lopez-Beltran, Francesco Massari, Matteo Santoni, Marina Scarpelli, Liang Cheng, Rodolfo Montironi

**Affiliations:** 1 Section of Pathological Anatomy, United Hospitals, School of Medicine, Polytechnic University of the Marche Region, Via Conca 71, 60126 Ancona, Italy; alessiacimadamore@gmail.com (A.C.); r.mazzucchelli@staff.univpm.it (R.M.); m.scarpelli@univpm.it (M.S.); 2 Department of Morphological Sciences, Cordoba University Medical School, 14071 Cordoba, Spain; em1lobea@gmail.com; 3 Medical Oncology, IRCCS Azienda Ospedaliero-Universitaria di Bologna; Via Albertoni n. 15, 40138 Bologna, Italy; fmassari79@gmail.com; 4 Oncology Unit, Macerata Hospital, Via Santa Lucia 2, 62100 Macerata, Italy; mattymo@alice.it; 5 Department of Pathology and Laboratory Medicine, Indiana University School of Medicine, Indianapolis, IN 46202, USA; liang_cheng@yahoo.com

**Keywords:** prostate cancer, Gleason grading system, grade groups, aggressive variant prostate cancer, DNA damage repair pathway, DNA mismatch repair, PARPi, immunotherapy

## Abstract

**Simple Summary:**

In 2021, the identification of effective biomarkers became a major focus of prostate cancer (PCa) in order to improve outcomes and select potentially responsive patients. The aim of this contribution is to review the main 2021 novelties in prognostic and therapeutic markers in PCa, with special reference to PCa grading, aggressive variant PCa and molecular markers predicting significant disease or response to therapy.

**Abstract:**

The 2021 novelties in prognostic and therapeutic tissue markers in patients with prostate cancer (PCa) can be subdivided into two major groups. The first group is related to prognostic markers based on morphological and immunohistochemical evaluations. The novelties in this group can then be subdivided into two subgroups, one involving morphologic evaluation only, i.e., *PCa grading*, and the other involving both morphologic and immunohistochemical evaluations, i.e., *aggressive variant PCa (AVPCa)*. Grading concerns androgen-dependent PCa, while AVPCa represents a late phase in its natural history, when it becomes androgen-independent. The novelties of the other major group are related to molecular markers predicting significant disease or response to therapy. This group mainly includes novelties in the molecular evaluation of PCa in tissue material and liquid biopsies.

## 1. Introduction

Prostate cancer is the most frequent malignant tumor in the male population worldwide, as well as one of the most common among all the leading causes of cancer-related death. Although several men receive an early-stage disease diagnosis and run an indolent course, many cases are characterized by locally advanced or metastatic disease at the time of diagnosis.

In 2021, the identification of effective biomarkers became a major focus in prostate cancer (PCa) in order to improve outcomes and select potentially responsive patients. The uropathologist has to integrate information from morphologic and immunohistochemical evaluations with the data obtained from molecular investigations. The goal is to improve the understanding of molecular data to identify actionable targets, as well as to develop novel treatments for patients with advanced PCa, and to extend survival [1].

The aim of this contribution is to review the main 2021 novelties in prognostic and therapeutic markers in PCa, with special reference to PCa grading, aggressive variant PCa and molecular markers predicting significant disease or response to therapy.

## 2. PCa Grading

The Gleason grading system is a quintessential prognostic factor when predicting findings in radical prostatectomy, biochemical failure, local recurrences, and lymph node or distant metastasis in patients receiving radical prostatectomy, radiation therapy and other therapies, including active surveillance. The grading system has been modified over time to reflect changes in diagnostic approaches, particularly a shift to an early detection of PCa. Modifications have been made to improve concordance between prostate biopsy and radical prostatectomy grading. This led to a precise description of the remaining three Gleason architectural patterns (i.e., grade 3–5) and a 5-tier grade grouping system (i.e., Grade Groups (GGs)) [2]. The latter was endorsed in the 2014 International Society of Urological Pathology (ISUP) conference [3]. The GGs correspond to groups of Gleason scores (GSs). They improve the communication between the morphologic observations and patients and clinicians. For instance, a GS 3 + 3 = 6 is assigned GG1 to indicate a favorable prognosis. A GS 3 + 4 = 7 is placed in a separate GG, i.e., GG2, compared to 4 + 3 = 7, i.e., GG3, to indicate a higher risk of recurrence of the latter.

### 2.1. ISUP and GUPS

The Gleason grading system was updated in 2019 following the ISUP consensus conference [4] and a Genitourinary Pathology Society (GUPS) “White Paper” [5]. The pros and cons of the ISUP and GUPS recommendations are reported in detail in an article on this topic [6]. There are some differences between the recommendations from the two societies, with some examples as follows.

**Figure 1:**
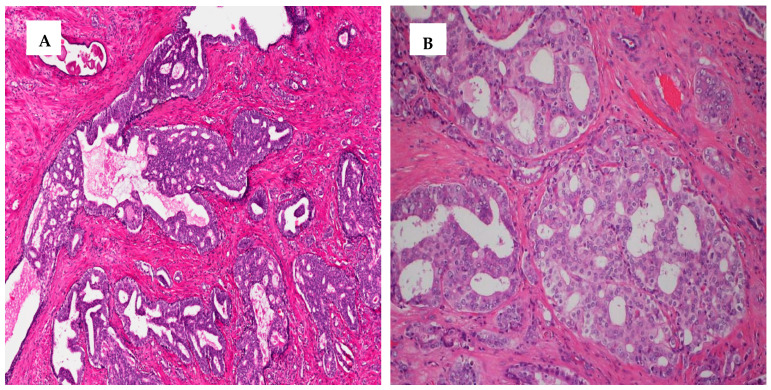
Intraductal carcinoma of the prostate (IDC-P) (10×) (**A**) and invasive cribriform PCa (**B**) (20×).

Intraductal carcinoma of the prostate (IDC-P) (Figure 1A) and invasive cribriform PCa (Figure 1B) are associated with worse outcomes.

The ISUP and the GUPS concur with the reporting of pure IDC-P, both recommending that pure isolated IDC-P should not be graded, and that immunohistochemistry should be performed cases when no associated invasive PCa component is seen.

There is no fundamental disagreement between the ISUP and the GUPS regarding the clinical implication of IDC-P associated with invasive PCa. Both societies agree that the presence of an IDC-P component in such a setting represents an adverse prognostic factor.

A major point of departure between the two societies is that ISUP recommends grading IDC-P when it is associated with invasive PCa, whereas GUPS recommends not grading IDC-P in any setting [6].

Concerning the issue of whether or not to grade IDC-P patterns into the overall grade being assessed, choosing one or the other recommendation “to apply uniformly in practice with fellow departmental or institutional colleagues, in consultation with local urologist, oncologist, and radiation oncologist stakeholders” has been suggested [7]. Which of the two sets of recommendations is used is “referenced in report comments, documenting the approach taken, especially for the key divergent scenario, where incorporating the pattern seen in extensive IDC-P, admixed with invasive carcinoma, into the grade assessment, or excluding it, could change the definitive GG” [7]. However, the current situation can be confusing for clinicians and patients as well.

Moving beyond the current definition and grading of IDC-P, its biological significance is the most important. Invasive cribriform and intraductal carcinoma are reported to have a significantly higher percentage of genome alterations (genomic instability) and somatic copy number alterations [8]. In 2018, Velho et al. analyzed 150 unselected patients with recurrent or metastatic prostate cancer and found that men with germline mutations were more likely to harbor intraductal/ductal histology (48% vs. 12%, *p* < 0.01) [9]. Recently, Lozano et al. examined the association of germline alterations in BRCA2 (*BReast CAncer gene 2*) patients and other tumor molecular features with IDC and/or cribriform histologies. Even though no significant differences were found between BRCA2 carriers and non-carriers in the prevalence of IDC, IDC was independently associated with bi-allelic BRCA2 alterations and phosphatase and tensin homolog gene (*PTEN*) homozygous loss [10].

Thus, the importance of recognizing the IDC-P growth pattern is not only related to the prognosis and management of the patient, but may have additional implications for the families and target therapy. To address this issue, the National Comprehensive Cancer Network^®^ (NCCN) guidelines for prostate cancer recommend considering germline testing for men who have prostate cancer with cribriform morphology or IDC [11,11].

### 2.2. Gleason Grading, Computational Pathology and Artificial Intelligence

Computer-based diagnosis of PCa on glass slides can be achieved by machine learning (ML) [8]. It is now possible to grade PCa on virtual slides, thus improving the reproducibility and accuracy of computer-based methods. Several researchers have dealt with PCa grading based on digital pathology and artificial intelligence (AI) [12]. Lucas et al. [13] have built an automatic classification of the Gleason patterns. When distinguishing between Gleason patterns 4 or higher and Gleason pattern 3, accuracy was 90%, with specificity and sensitivity being 94% and 77%, respectively. The concordance between their computer-based GG evaluation and the evaluation made by a pathologist was 65%. This method can assist pathologists in defining GG in prostate biopsies.

AI can create algorithms that allow a generalist to function as a specialist. Nagpal et al. [14] adopted “a supervised learning method to develop a deep learning system (DLS) for PCa grading” on radical prostatectomies. The accuracy of the GG assignment was assessed by generalists, in comparison to specialists. The DLS outperformed the generalist pathologists.

## 3. Aggressive Variant of PCa

PCa may evolve into an androgen receptor (AR)-independent phenotype, characterized by a rapidly progressive disease course, including secondary deposits in visceral sites [15]. The term aggressive variant prostate cancer (AVPCa) is used to refer to this clinically aggressive form [15]. It shows low or absent androgen receptor (AR) expression and is associated with low serum levels of prostate-specific antigen (PSA) [16]. Transformation to AR-independent AVPCa, i.e., metastatic castration-resistant prostate cancer (mCRPC), occurs as a mechanism of adaptive resistance to AR-targeted therapies, including newer AR-targeted treatments [16]. Mutations and amplifications of the AR gene are reported in 1% of primary PCa and in approximately 60% of metastatic tumors. AR mutations predominantly occur in the AR androgen-binding domain [17,18]. Some mutations enable the activation of AR by other adrenal androgens, some increase AR-transcriptional activity, while others have been demonstrated to confer resistance to enzalutamide, abiraterone and other anti-androgens drugs [19].

**Figure 2:**
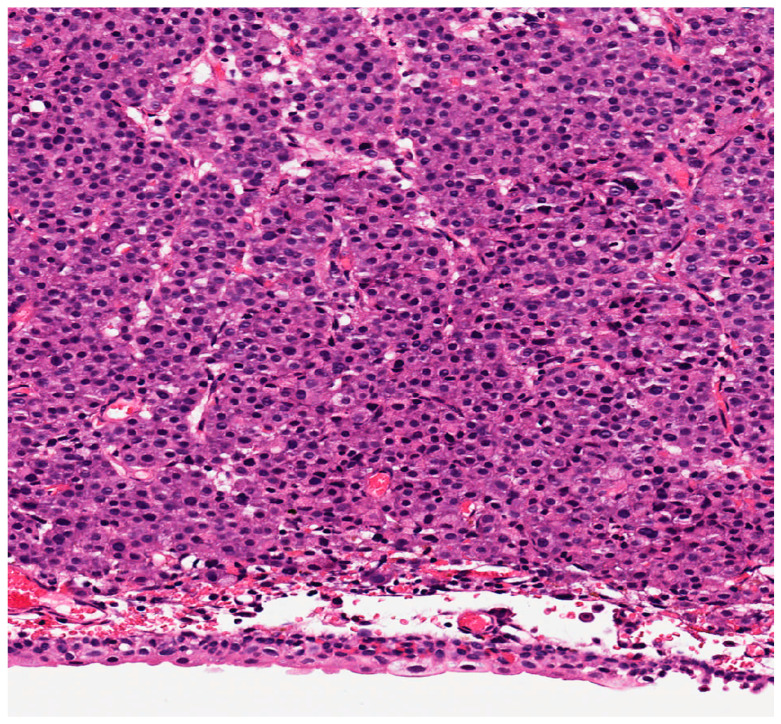
Aggressive variant PCa (10×).

Morphologically, AVPCa is made up of solid sheets of cells devoid of pleomorphism, with round and enlarged nuclei with prominent nucleoli and slightly basophilic cytoplasm [20] (Figure 2). The cells do not show the typical architectural features of prostatic adenocarcinoma, and mimic the undifferentiated carcinoma of other organs and locations. The final diagnosis is based on the immunohistochemical expression of markers usually seen in PCa, particularly the Prostate-Specific Membrane Antigen (see below) [11].

As shown with immunohistochemical techniques, a subset of AVPCa can also express neuroendocrine (NE) makers, such as chromogranin A, synaptophysin and CD56. Since such tumors can develop following androgen receptor pathway inhibition, castration-resistant PCa transdifferentiates in a clonally divergent manner to become a treatment-related NE PCa. This lineage plasticity program is dependent on activation of the transcription factor E2F1 in concert with the BET bromodomain chromatin reader BRD4. Thus, blocking this E2F1/BRD4-regulated program with BET inhibitors decreased the growth of neuroendocrine PCa tumor models [21]. In this setting, a study on CRPC and neuroendocrine PCa models has demonstrated that combination therapy with PARP inhibitors and CDK4/6 inhibitors resulted in synergistic suppression of the p-Rb1-E2F1 axis and induced apoptosis and suppressed neuroendocrine differentiation in PCa, paving the way for new trials testing this combination [22].

The tumors that do not show NE differentiation might harbor somatic and/or germline alterations in the DNA repair pathway [20]. The identification of such subtypes has an important clinical relevance for the potential benefits of platinum-based chemotherapy, poly (ADP-ribose) polymerase inhibitors (PARPi), and further therapies [15] (see below).

## 4. Molecular Markers Predicting Significant Disease or Response to Therapy

Morphologic and immunohistochemical findings have been useful in predicting tumor behavior and prognosis. Recent investigations have demonstrated that molecular biomarkers can predict clinical outcomes in a manner that outperforms traditional morphology [23]. Many prognostic biomarker candidates have been proposed [14] (Table 1). For instance, several investigations have shown that the loss of *PTEN* is linked to poor prognosis in PCa patients [24]. In a cohort of mCRPC, PTEN deletions were reported in around 30% of patients and another 10% of patients harbored truncating mutations and gene fusions. PTEN loss is typically mutually exclusive with several other genomic alterations in human prostate cancer, including SPOP mutation and CHD1 loss [25,26]. Its loss of expression in biopsy samples predicts increased risk of CRPC, metastasis, and prostate-cancer-specific mortality in surgically treated patients [26,27]. As well as being a prognostic biomarker, PTEN loss has proved to be a predictive biomarker of response to therapy with Ipatasertib [28]. Ipatasertib is a novel oral ATP-competitive inhibitor of Akt that, in combination with abiraterone, has been demonstrated to be superior to abiraterone alone in patients with mCRPC, especially in tumors with PTEN-loss. This result was recently confirmed at ASCO 2021 by the results of the phase III trial (IPATential150). Improved radiographic progression-free survival (rPFS) was reported for the combination of Ipatasertib plus Abiraterone plus prednisone compared to placebo plus abiraterone plus prednisone as a first-line therapy in patients with PTEN-loss, but not in the intention to treat (ITT) group. In both, trial PTEN loss was defined by immunohistochemistry (IHC) as a minimum of 50% of the specimen’s tumor area with no detectable PTEN staining (by Ventana assay using SP218 antibody). During exploratory biomarker analysis, the authors evidenced that a consistent benefit in the Ipatasertib arm was observed when PTEN loss was reported in a higher percentage of tumor area. Moreover, patients with genomic alterations in PIK3CA/AKT1/PTEN assessed by Next Generation Sequencing (NGS) had a greater rPFS advantage in the combination arm compared to patients with no detectable alterations [29].

Genomic loss can also be determined by fluorescence in situ hybridization (FISH) techniques. Immunohistochemistry and FISH are often concordant, although some cases of PTEN expression at IHC may report deletion at FISH [27]. PIK3CA/AKT1/PTEN alterations can also be detected by liquid-biopsy-based essay. However, the NGS assay used should be designed to reveal deletions and rearrangements in the target genes. In the recently published study by Tukachinsky et al. on the genomic analysis of circulating tumor DNA (ctDNA) in 3334 patients with advanced PCa, only 9% of patients reported an alteration in this pathway because the platforms used in the study were not designed to detect deletions, leading to marked under-detection [30].

RNA-based analyses have been developed in order to guide management decisions for very low-risk and low-risk primary PCa. The role of such RNA-based assays in comparison with well-established prognostic markers in the active surveillance setting, for instance, remains to be proved [31].

### 4.1. Mutations to the DNA Damage Repair Pathway and PARPi

Advances in the technology of DNA sequencing have clarified the genomic landscape of PCa, including AVPCa [11]. Somatic mutations may change over time, due to selective pressure from genetic instability and therapy. Repeat testing of tumor DNA is needed during the course of the disease. Testing from primary or metastatic tissues or blood may help guide treatment options [32,33]. Tumor-based testing has the potential to identify germline mutations, with implications for the predisposition to inherited PCa.

Up to 20–25% of metastatic PCa harbor germline or somatic changes in DNA repair genes involved in the pathways of mismatch repair (MMR) and homologous recombination repair (HRR) [32,33]. HRR defects can predict responses to PARPi, an enzyme that is involved in alternative DNA repair mechanisms. PARPi therapy has shown an improved overall survival in metastatic-castration-resistant PCa in patients with somatic and/or germline alterations in HRR genes [34]. However, the effect of PARPi therapy is not the same for all individuals with HRR gene mutations. In fact, in the exploratory gene-by-gene analysis of the PROFOUND trial, the best objective response rate (ORR) was reported for BRCA-mutated patients with a significant difference in overall survival (OS) (20.1 vs. 14.4 months for olaparib and enzalutamide or abiraterone, respectively), while no difference in OS was reported in patients with ATM or CDK12 alterations [35]. At present, NCCN, ESMO and EAU guidelines recommend tumor testing for HRR for all metastatic patients [11,36,37,38]. However, information such as which type of test, how many genes and which type of alterations are necessary to perform is not specified in these guidelines. A recent evaluation of the cost-effectiveness of genomic tests to identify patients that could benefit from olaparib-therapy evidenced that a genomic test restricted to BRCA1,BRCA2, and ATM is preferred over the standard care strategy, directed to test all 15 prespecified genes [39].

### 4.2. Mutations Other than Defective DNA Repair Mechanisms

Germline mutations in the mismatch repair (MMR) genes (i.e., MLH1, PMS2, MSH2, and MSH6) are seen in Lynch syndrome, an inherited condition that predisposes individuals to an increased risk of developing many different types of cancer. Various publications have suggested a slight increase in risk for PCa in men with this syndrome [40]. HOXB13 G84E is a germline variant associated with increased risk of developing PCa [41]. It is not clear whether it is associated with an increased disease aggressiveness.

### 4.3. Molecular Markers and Immunotherapy

**Figure 3:**
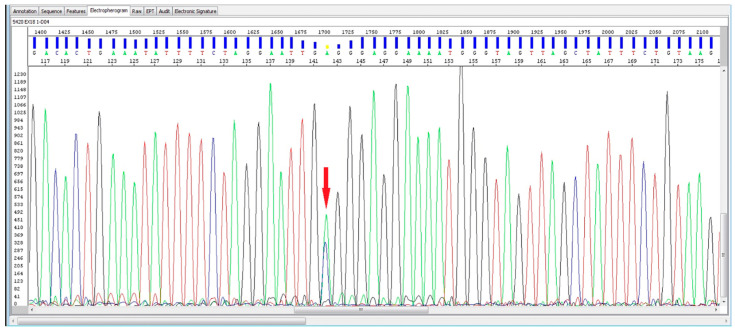
BRCA2 gene mutation in AVPCa (red arrow indicates the BRCA2 defect).

A defective DNA repair system can increase the frequency of DNA mutations. This is a very important observation in the development of the antitumor immune response. Mutations in the *BRCA2* gene (Figure 3) have been observed in melanoma patients with a better response to anti-PD-1 (programmed cell death-1 ligand 1) therapy [32]. Such findings provide support to the potential use of the mutation status in DNA repair genes, in order to predict the response to immunotherapy in AVPCa.

Immune checkpoint inhibitors have been approved in advanced solid tumors harboring genetic defects in DNA mismatch repair (dMMR), including PCa. The detection of dMMR, using either immunohistochemistry or molecular methods, is therefore needed in patients with advanced castration-resistant PCa who are being considered for immunotherapy [42].

However, human PCa specimens are mostly immunologically cold tumors and do not respond well to immunotherapy, due to their complex immune evasion mechanisms. Bou-Dargham et al. identified eight different immune evasion clusters, in which the majority of the clustered PCa patients (around 90%) exhibited immunological ignorance that can result from the absence of tumor-specific antigens that activate the immune system, or from the failure of antigen-presenting cells to recognize cancer antigens. Interestingly, they also reported a series of biomarkers that could predict responses to various immunotherapies CD48, SP140, KIRREL, RHOB, FBXO17, ANAPC1, EGFR, SOCS3, ALOX15, and UBR2 [43].

### 4.4. Molecular Markers and Liquid Biopsy

The analysis of circulating tumor cells, circulating free DNA and exosomes in liquid biopsy has a fundamental role in diagnosis, prognosis and treatment planning for PCa patients. For instance, somatic alterations can be assessed by extracting DNA from a tumor tissue sample or using circulating tumor DNA (ctDNA) extracted from a plasma sample [44]. Peripheral blood samples are typically used for the germline mutation analysis test using the DNA extracted from peripheral blood leucocytes. The main advantages of this technique, compared to the tissue test, are that liquid biopsy is an easily repeatable and non-invasive test, which represents tumor heterogeneity better than primary biopsy, and is also better at capturing changes and/or resistance mutations in the genetic tumor profile during disease progression. Furthermore, ctDNA can provide information on mutation status and guide treatment options in patients with AVPCa. Clinical validation and test implementation into routine clinical practice are currently limited [45].

Urinary exosomes, vesicles with a lipid bilayer membrane enclosing proteins, lipids, RNA, and DNA have been thought to originate from cells of the urogenital tract and constitute a source of potential biomarkers for PCa, such as PCA3, and TMPRSS2:ERG, which are currently included in a diagnostic test [46,47]. Recently, Dhondt et al. developed a mass-spectrometry-based proteomic analysis of urinary EV that identifies a unique biological profile in prostate cancer, which is not uncovered by the analysis of soluble proteins [48].

PCa is primarily driven by AR signaling. This has been exploited from the therapeutic perspective by applying receptor blockade and/or androgen withdrawal [49]. The development of constitutively active AR splice variants is one of the mechanisms involved in hormonal therapy resistance in AVPCa. The detection of one such variant (i.e., AR splice variant Arv7) in circulating tumor cells or in peripheral whole blood, without the need for CTC capture, can predict poor response to abiraterone and enzalutamide, i.e., second-generation AR antagonists [50,51]. Tissue-based ARv7 detection by immunohistochemistry or in situ hybridization remains at an experimental level.

The radiopharmaceutical Radium-223 (Ra-223) improves survival and prevents skeletal-related events in men with mCRPC. However, the molecular determinants of response are poorly understood, and there is an unmet clinical need for biomarkers to guide the use of Ra-223. Circulating tumor cell (CTC) analyses may serve as prognostic and/or predictive biomarkers for Ra-223 response. The pre-treatment digital CTC RNA score was significantly higher in patients who demonstrated a progression on bone scans compared to those with stable or decreased disease burden [52]. Radium-223 causes double-strand DNA breaks and produces γH2AX, a potential biomarker for response. In a study by Chatzkel et al., the feasibility of tracking γH2AX positivity and numeration in circulating tumor cells was shown [53].

Circulating tumor cells (CTCs) from men with metastatic PCa express immune checkpoint ligands B7-H3, PD-L1, PD-L2, and CTLA-4 in a heterogenous manner, and the detection of such immune checkpoints may enable monitoring patients on immunotherapy [54].

### 4.5. Antibody-Drug Conjugates

Antibody-Drug Conjugates (ADCs) are novel compounds consisting of cytotoxic agents linked to specific antibodies that are able to recognize antigens expressed over cancer cells’ surfaces. Researchers are focusing on PSMA, STEAP1, TROP2, CD46 and B7-H3 as optimal antigens that may be targeted by ADCs (Table 2) [55].

**Figure 4:**
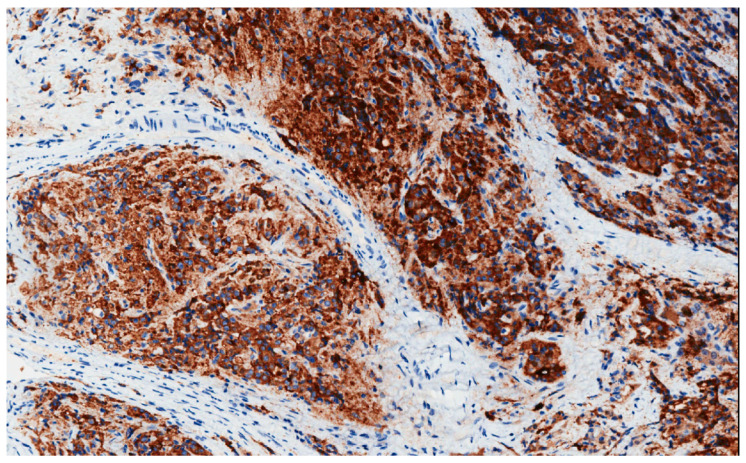
Immunohistochemical expression of PSMA in an androgen independence PCa (20×).

Prostate-specific membrane antigen (PSMA), with folate hydrolase, carboxypeptidase and internalization activities, is an example of these targets. It is expressed in the epithelial cells in the prostate and strongly upregulated in PCa, with elevated expression correlating with progression, metastasis and androgen independence (Figure 4). Recently, PSMA has been an active target of investigation by several approaches, including the utilization of small molecule inhibitors, RNA aptamer conjugates, PSMA-based immunotherapy, and PSMA-targeted prodrug therapy. On March 2021, a phase 1 study began to enroll mCRPC patients to evaluate ARX517, a PSMA ADC conjugated to microtubule-disrupting toxins AS269 (NCT04662580) [55,56].

## 4. Conclusions

The Gleason grading system is a powerful marker for predicting biochemical recurrence, secondary deposits and survival. The Gleason grading system was updated in 2019 by the ISUP and GUPS [4,5]. A major point of departure between the two societies is that the ISUP recommends grading IDC-P when associated with invasive PCa, whereas GUPS recommends not grading IDC-P in any setting [6]. Tissue samples may show the morphologic spectrum and features of AVPCa. In particular, immunohistochemistry for PSMA, AR signaling markers and classic NE markers offer support to the definition of the subtypes [11]. Recent investigations have shown that tissue material and liquid biopsy from patients with AVPCa, including those with mCRPC, can provide clinically relevant molecular information, including somatic mutations to the DNA damage repair pathway and mutations other than defective DNA repair mechanisms. This has important clinical relevance for the potential benefits of platinum-based chemotherapy, PARPi, and further therapies. The incorporation of tissue and genetic biomarkers into current PCa prediction models, in a process called information fusion, will optimize future decision-making and improve patient outcomes [32]. However, some issues are still the objects of debate among the scientific community. How to identify patients who can benefit from a target therapy, which type of test to offer, test timing and others are questions that need to be solved in future years.

## Figures and Tables

**Table 1:** Tissue biomarkers in predicting upgrading and/or significant disease in prostate cancer (IDC-P: prostatic intraductal carcinoma).

Biomarkers	Description	Clinical Impact
*PTEN*	*PTEN* loss is associated with adverse oncological outcomes	By itself or in combination with other biomarkers helps distinguish indolent tumors from those likely to progress
*APC, RASSF1, TBX15 methylation*	DNA methylation biomarkers associated with cribriform architecture and IDC-P	Detection of DNA methylation-based biomarkers may act as indicators of cribriform and/or IDC patterns in biopsy tissue samples
*SChLAP 1*	Long non-coding RNA overexpressed in 25% of PCa	High expression significantly correlates with metastatic progression of PCa
*BRCA 2*	*BRCA2*-mutant PCa harbor increased genomic instability and a mutational profile similar to metastatic rather than localized disease	*BRCA2*-mutant PCa are uniquely aggressive, often occur in young men, have higher rates of lymph node and distant metastasis, and increased mortality, justifying aggressive initial treatment

**Table 2:** The clinical trial data of ADCs in PCa. ARSI = Androgen Receptor Signaling Inhibitor; mCRPC = metastatic Castration-Resistant Prostate Cancer.

Tissue Target	Drug	Disease	Strategy	Phase	Estimated Completion Date	NCT Number
PSMA	ARX517	mCRPC	Single agent	1	August 2024	NCT04662580
STEAP1	AMG 509	PCa refractory to a novel antiandrogen therapy and not more than 2 taxane regimens	Single agent	1	October 2025	NCT04221542
TROP2	Sacituzumab govitecan (IMMU-132)	mCRPC progressing on ARSI	Single agent	2	October 2021	NCT03725761
CD46	FOR46	mCRPC	Single agent	1	December 2021	NCT03575819
B7-H3	MGC018	Advanced solid tumors including prostate cancer	Single agent or with anti-PD-1 antibody MGA012	1/2	May 2025	NCT03729596

## Data Availability

No new data were created or analyzed in this study. Data sharing is not applicable to this article.
